# Small bowel diverticulum causing primary enterolithiasis: a rare diagnosis with definitive surgical intervention

**DOI:** 10.1093/jscr/rjaf037

**Published:** 2025-02-05

**Authors:** Angel Guan, Kendall Vignaroli, Kevin Perez, So Un Kim, Danielle Cremat, Aldin Malkoc, Jasmine Lam, Ngoc Patrick Van Nguyen

**Affiliations:** The Division of General Surgery, Department of Surgery, Arrowhead Regional Medical Center, 400 N Pepper Ave, Colton, CA 92324, United States; The Division of General Surgery, Department of Surgery, Arrowhead Regional Medical Center, 400 N Pepper Ave, Colton, CA 92324, United States; The Division of General Surgery, Department of Surgery, Arrowhead Regional Medical Center, 400 N Pepper Ave, Colton, CA 92324, United States; The Division of General Surgery, Department of Surgery, Arrowhead Regional Medical Center, 400 N Pepper Ave, Colton, CA 92324, United States; The Division of General Surgery, Department of Surgery, Arrowhead Regional Medical Center, 400 N Pepper Ave, Colton, CA 92324, United States; The Division of General Surgery, Department of Surgery, Arrowhead Regional Medical Center, 400 N Pepper Ave, Colton, CA 92324, United States; The Division of General Surgery, Department of Surgery, Arrowhead Regional Medical Center, 400 N Pepper Ave, Colton, CA 92324, United States; The Division of Colorectal Surgery, Department of Surgery, Kaiser Permanente Fontana Medical Center, 9961 Sierra Ave., Fontana, CA, United States

**Keywords:** enterolithiasis, small bowel obstruction, gallstones, obstructions

## Abstract

Primary enterolithiasis is characterized by the formation of stones within the small bowel. The prevalence is estimated to be ~0.3% to 10% in selected populations. Due to its rarity, diagnosis is often delayed. We present the case of a 77 year old male who presented with small bowel obstruction, which was initially thought to be due to intussusception seen on abdominal computed tomography scan. He underwent two diagnostic laparoscopies within 1 month because his small bowel obstruction did not resolve with the initial surgery. The primary enterolith was not discovered until the second surgery where a 5 cm primary enterolith was seen in the small bowel causing early mucosal necrosis. The stone was removed, and the enterotomy was closed. After the enterolith was removed, the patient’s upper gastrointestinal symptoms completely resolved.

## Introduction

Enterolithiasis, a rare cause of small bowel obstruction, is the formation of gastrointestinal concretions that develop in the setting of intestinal stasis. Risk factors for enterolithiasis include intestinal diverticula, surgical enteroanastomoses, blind pouches, afferent loops, incarcerated hernias, small intestinal tumors, intestinal kinking from intra-abdominal adhesions, stenosing, or stricturing Crohn’s disease, and intestinal tuberculosis [1–3]. Enterolithiasis is classified into primary and secondary types, with secondary being most commonly from gallstone ileus or nephrolithiasis [4–5]. We discuss the roles of careful preoperative assessment, surgical management, and the importance of the diagnosis and surgical management of primary enterolithiasis.

## Case presentation

**Figure 1:**
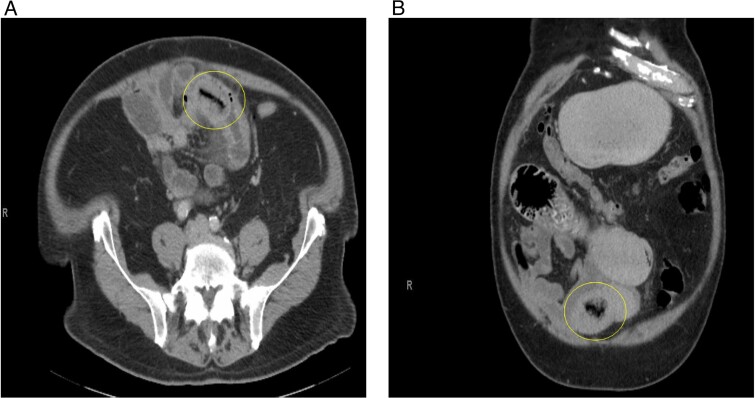
(A) Initial CT abdomen (transverse view) revealing what was thought to be intussusception but turns out to be primary enterolith with an air filled core. (B) Initial CT abdomen (coronal view) revealing what was thought to be intussusception but turns out to be primary enterolith with an air filled core.

A 77-year-old male with a medical history of hyperlipidemia presented to the emergency department (ED) with diffuse lower abdominal pain. Physical examination revealed mild tenderness in the lower left quadrant. Computed tomography (CT) of the abdomen with oral contrast revealed an abnormally thickened small intestinal loop with possible intussusception causing low-grade partial obstruction (Fig. 1). Due to increasing abdominal pain, the patient underwent diagnostic laparoscopy, and a narrowed segment of the small bowel was found. Intussusception was not seen, the scarred and narrowed segments were resected, and primary stapled anastomosis was performed. The distal collapsed bowel was examined 2 feet from the transition point, and no lesions or masses were observed. The pathology of the resected bowel segment was benign.

**Figure 2:**
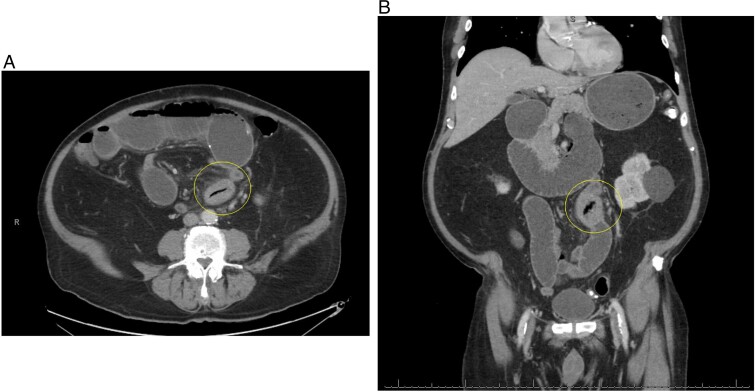
(A) Repeat CT scan (transverse view) on readmission revealing the same primary enterolith with the core pit that mimics intussusception. (B) Repeat CT scan (coronal view) on readmission revealing the same primary enterolith with the core pit that mimics intussusception.

**Figure 3:**
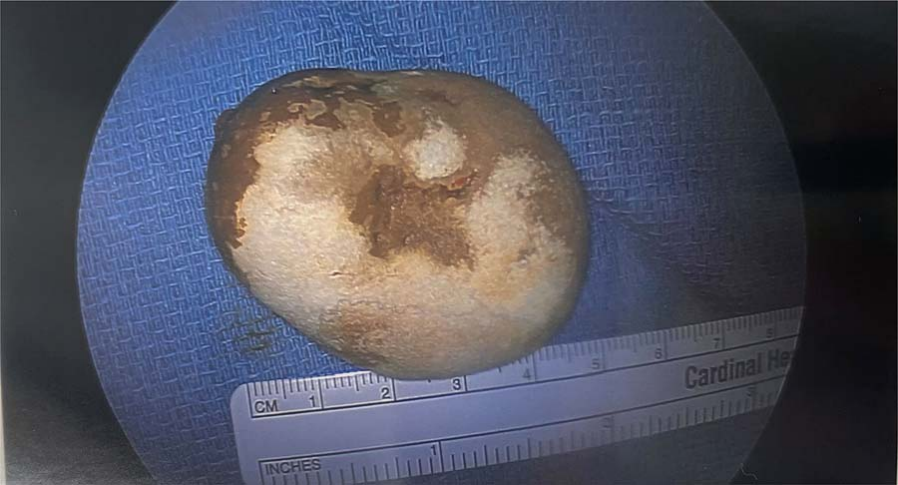
Round primary enterolith extracted from the second diagnostic laparoscopy. The removal of this stone lead to full resolution of GI symptoms for this patient.

The postoperative course was complicated by ileus, which did not resolve until postoperative day 11. The patient was discharged in stable condition and returned to the hospital 3 days after with oral administration intolerance. At this time, abdominal examination revealed significant abdominal distention. Incisions were healed and non-tender. A repeat abdominal CT scan showed dilated fluid-filled loops of the small bowel consistent with high-grade small bowel obstruction and focal intussusception of a loop of the small bowel distal to the small bowel anastomosis with associated wall thickening (Fig. 2). Over the next two weeks, the patient had a tumbling small bowel obstruction. His abdominal symptoms improved and worsened cyclically. A small bowel follow through was obtained and showed contrast within the colon in 45 min, signifying partial and incomplete obstruction. Esophagoduodenoscopy revealed two duodenal diverticulum. On hospital Day 13 during the patient’s second admission, he developed severe abdominal pain and was taken back to the operating room for a second diagnostic laparoscopy, which revealed an inflamed small bowel distal to the previous fully healed small bowel anastomosis. After manual palpation, we observed a hard mobile mass within the small bowel. An enterotomy was performed, and a 5  × 4 cm calcified stone appeared to cause pressure necrosis of the small bowel mucosa. The stone was round and without any facets (Fig. 3). Primary repair of the small bowel was then performed. The entire small bowel run from the ligament of Treitz to the cecum, and no additional stones or pathology was palpated except for a small 1.5 cm meckel diverticulum ~2 feet proximal to the ileocecal junction. The patient recovered well after surgery. Abdominal bloating and pain resolved, and the patient began tolerating the diet by postoperative day 2. The patient was seen as an outpatient in the clinic and has been doing well for 3 months after surgery.

## Discussion

Primary enterolithiasis pathophysiology can be subcategorized into true and false enteroliths [5–6]. True enteroliths are made of substances that form into chyme, which subsequently form either choleic acid or calcium stones. False enteroliths are made from insoluble substances in the bowel, such as bezoars, insoluble solvents, and water-suspended insoluble salts. Proximal primary enteroliths are composed of choleic acid salts and distal enteroliths are calcified [7]. Clinical presentation includes ‘tumbling’ gastrointestinal (GI) symptoms which include abdominal pain, distention, nausea, and vomiting of occasionally sudden but often fluctuating subacute nature, which occurs because of the enterolith tumbling through the bowel lumen. Thorough history and physical examination coupled with radiologic imaging help establish a diagnosis in at-risk patients. Complications include bowel obstruction, direct pressure injury to the intestinal mucosa, intestinal gangrene, intussusceptions, afferent loop syndrome, diverticulitis, iron deficiency anemia, gastrointestinal hemorrhage, and perforation [8]. Mortality of primary enterolithiasis may reach 3%, and secondary enterolithiasis 8% [9]. Risk factors include poorly conditioned patients with significant obstruction and delay in diagnosis [10]. Treatment relies on timely recognition of the disease and endoscopic or surgical intervention.

Our case is an example of a rare cause of small bowel obstruction that caused ‘tumbling’ GI symptoms. The patient’s symptoms persisted after the initial surgery because the enterolith had not been discovered and removed. Patients with primary enteroliths usually receive delayed care because of the rarity of the pathology. The important history and physical findings in this patient care revealed his tumbling GI symptoms. It is possible the ‘intussusception’ noted on imaging was the primary enterolith with an air-filled pit disguising the stone as gas within small bowel. It is likely that this patient developed the primary enterolith from his diverticulum in the duodenum and Meckel diverticulum, causing stasis within the small bowel and insoluble solvents to precipitate in these areas. This rare and unique case discusses the possibility of primary enteroliths, the importance of the patient’s history, and the physical and treatment options.
